# Metabolomic approach for a rapid identification of natural products with cytotoxic activity against human colorectal cancer cells

**DOI:** 10.1038/s41598-018-23704-9

**Published:** 2018-03-28

**Authors:** Vittoria Graziani, Monica Scognamiglio, Valentina Belli, Assunta Esposito, Brigida D’Abrosca, Angela Chambery, Rosita Russo, Marta Panella, Aniello Russo, Fortunato Ciardiello, Teresa Troiani, Nicoletta Potenza, Antonio Fiorentino

**Affiliations:** 1 Dipartimento di Scienze e Tecnologie Ambientali Biologiche e Farmaceutiche (DiSTABiF), Università degli Studi della Campania “Luigi Vanvitelli”, via Vivaldi 43 I-, 81100 Caserta, Italy; 2 Max Planck Institute for Chemical Ecology - Beutenberg Campus, Hans-Knöll-Straße 8 D-, 07745 Jena, Germany; 3 Dipartimento di Internistica Clinica e Sperimentale “Flaviano Magrassi”, Università degli Studi della Campania “Luigi Vanvitelli” Via Pansini, 5 -, I-80131 Napoli, Italy

## Abstract

The discovery of bioactive compounds from natural sources entails an extremely lengthy process due to the timescale and complexity of traditional methodologies. In our study, we used a rapid NMR based metabolomic approach as tool to identify secondary metabolites with anti-proliferative activity against a panel of human colorectal cancer cell lines with different mutation profiles. For this purpose, fourteen Fabaceae species of Mediterranean vegetation were investigated using a double screening method: ^1^H NMR profiling enabled the identification of the main compounds present in the mixtures, whilst parallel biological assays allowed the selection of two plant extracts based on their strong anti-proliferative properties. Using high-resolution 2D NMR spectroscopy, putative active constituents were identified in the mixture and isolated by performing a bio-guided fractionation of the selected plant extracts. As a result, we found two active principles: a cycloartane glycoside and protodioscin derivative. Interestingly, these metabolites displayed a preferential anti-proliferative effect on colon cancer cell lines with an intrinsic resistance to anti-EGFR therapies. Our work provides an NMR-based metabolomic approach as a powerful and efficient tool to discover natural products with anticancer activities circumventing time-consuming procedures.

## Introduction

The development of plant-derived substances has established the basis of sophisticated traditional medicine and, nowadays, plants still continue to be an essential source of pharmaceutical agents^1,2^. In particular, natural products have been the pillar of cancer chemotherapy for several years. Even though combinatorial chemistry provides a wide range of new and synthetic drugs, natural products are likely to provide lead compounds for the development of novel agents with enhanced biological properties^3,4^.

This success in drug discovery is linked to the high chemical diversity of natural sources; nevertheless, the chemical variability and the vast number of metabolites, which occur in the plant kingdom, make the detection and characterisation processes challenging to perform^5^. Traditional methodologies to discover new active molecules have different drawbacks, such as their complexity and inherent lengthy timescales, given that this research requires different separation and isolation steps before an active compound will emerge from the crude extract. Recently, the screening of plant-derived mixtures has become an effective way for rapid selection of metabolites with biological properties^6,7^.

In this scenario, metabolomics can provide a pertinent and convenient strategy, by allowing the direct study of the crude extract, simultaneously observing a wide range of metabolites belonging to different chemical classes, without the need of time-consuming isolation procedures^8^. The rapid sample preparation, shorter NMR measurement times, advanced data analysis methods and the use of advanced two-dimensional (2D) techniques to determine the structures of known or unknown compounds, make the NMR-based metabolomic approach extremely powerful for the profiling of crude extracts and the rapid identification of natural products^9^. Furthermore, relating putative biological activities with metabolomics is feasible by integrating chemometric methods with bioactivity results^10,11^. In this work, we combined an NMR-based metabolomic approach with MTT (3-[4,5-dimethylthiazol-2-yl]−2,5-diphenyltetrazolium bromide) tetrazolium salt colorimetric assays to select plant species belonging to Fabaceae family with anti-proliferative properties against a panel of genetically different colon cancer cell lines.

Colorectal cancer is one of the most frequently diagnosed malignant diseases in Europe and one of the leading causes of cancer-related death worldwide^12^. Although the outcome of patients with metastatic colorectal cancer (mCRC) has improved during the last years, current therapies available are not sufficiently effective^13^ and their efficacy is limited by the side effects of the drugs and/or the development of resistance^14,15^. For these reasons, there is a resurgence of interest for natural products, such as medicinal plants and dietary means as an alternative solution to cure mCRC patients. In the plant kingdom, the Fabaceae family represents a heritage of high biodiversity for their richness in secondary metabolites^16^, particularly significant for human health, either as dietary supplements or as pharmacological agents^17^. Furthermore, many studies reveal an interesting correlation between Fabaceae activities and colon cancer prevention and therapy, identifying this family as a promising source of new effective molecules against colon cancer. Soy (*Glycine max*) contains many bioactive compounds, including isoflavones. Epidemiological studies have shown that a high-level intake of soy-derived products contributes to a lower incidence of colorectal cancer in Asian countries. Among various soy isoflavones, genistein (4,5,7-trihydroxyisoflavone) (GEN) has been evaluated as a good candidate in colon cancer prevention^18^. The formononetin, constitutive isoflavones from barrel medic (*Medicago truncula*), exerts strong antiangiogenic effects reducing colon cancer proliferation both *in vitro* and *in vivo* experiments^19^. A naturally occurring rotenoid deguelin, isolated from *Mondulea sericea*, an African legume species, significantly inhibited IL-8 gene expression, induces apoptosis in colon cancer cells by down regulating the NF-kB signalling^20^. Isoliquiritigenin, a chalcone from roots of liquorice roots (*Glycyrrhiza glabra*) could be a promising adjuvant in colon cancer treatment, reducing tumour growth and protecting kidney and liver against chemotherapy-induced toxicity in a mouse xenograft model of colon carcinoma^21^. The triterpenoid B-group soyasaponins have been found to induce macroautophagy in human colon cancer cells^22^.

Thus, in the present work we applied a new screening method to seek secondary metabolites from fourteen Mediterranean Fabaceae species, which could be promising lead-structures to develop novel agents in CRC chemotherapy.

## Results

### Metabolic profiling of the selected Fabaceae species

The selected plants *(Astragalus boeticus* L*., Lathyrus cicera* L.*, Lathyrus clymenum* L.*, Medicago minima* (L.) L., *Melilotus neapolitanus* Ten.*, Ononis variegata* L., *Pisum sativum* L. subsp. *biflorum* (Raf.) Soldano, *Trifolium campestre* Schreb.*, Trifolium cherleri* L.*, Trifolium scabrum* L. subsp. *scabrum, Trigonella esculenta* Willd., *Vicia bithynica* (L.) L.*, Vicia pseudocracca* Bertol.*, Vicia angustifolia* L.) were analysed for their metabolic phenotype by multinuclear NMR spectroscopy. ^1^H NMR profiling of these plant extracts allowed the identification of the main compounds present in the mixture. The metabolite assignment was done by comparing peak chemical shifts to those found in literature^23^ and in Human Metabolome Database (HMDB), and furthermore, all the metabolite structures were confirmed by 2D NMR experiments. The peculiar NMR values of 31 identified metabolites were reported in Table S1. This analysis revealed that all the studied species had a similar composition in primary metabolites (amino acids, organic acids and sugars) but a high variability was seen in terms of secondary metabolites, the majority of which were phenols. A group of compounds well represented in all the analysed species are flavonoids, characterised by distinctive doublets in the aromatic region of the proton spectra. Among these, quercetin, kaempferol and their derivatives were the most common compounds. Other flavonoids, on the contrary, were only present in selected species: a C6-glycosylated apigenin derivative and catechins were found in *T. campestre* and *L. clymenum*, respectively; meanwhile, in the *M. minima*.^1^H-spectrum different doublets in the region between 6.30 and 7.50 ppm supported the presence of isoflavone compounds such as daidzein, daidzin and genistein.

Cinnamic acid derivatives and caffeic acid were also widespread in all the studied plants. Chlorogenic acids were found in *V. bithynica*, *V. angustifolia*, while coumarin and their glucoside precursors such as *cis*/*trans*-melilotoside and dihydromelilotoside were detected in *M. neapolitanus*^24^. At lower fields, the singlet at δ_H_ 9.15 and the triplet at δ_H_ 8.86 indicated the presence of trigonelline, a methylbetaine derivative of nicotinic acid. In the opposite region of spectra, signals of terpene metabolites were detected in *A. boeticus*. In detail, two doublets at δ_H_ 0.37 (J = 4.6 Hz) and δ_H_ 0.57 (J = 4.6 Hz) revealed the presence of cycloartane compounds in this plant extract.

### Cytotoxic activity of plant extracts

The potential anti-proliferative activity of the plant extracts studied here was evaluated against three human colorectal cancer cell lines (Caco-2, HT-29 and HCT-116), which were selected for their diverse mutation profiles and their resulting different medical aspects. In particular, Caco-2 cell line has no genetic alterations in KRAS, NRAS, BRAF and PIK3CA genes, which were known to be associated with intrinsic resistance to anti-EGFR therapies, such as cetuximab and panitumumab. On the contrary, HT-29 and HCT-116 cells displayed a different genetic profile, harbouring BRAF V600E and KRAS/PIK3CA, respectively.

**Figure 1:**
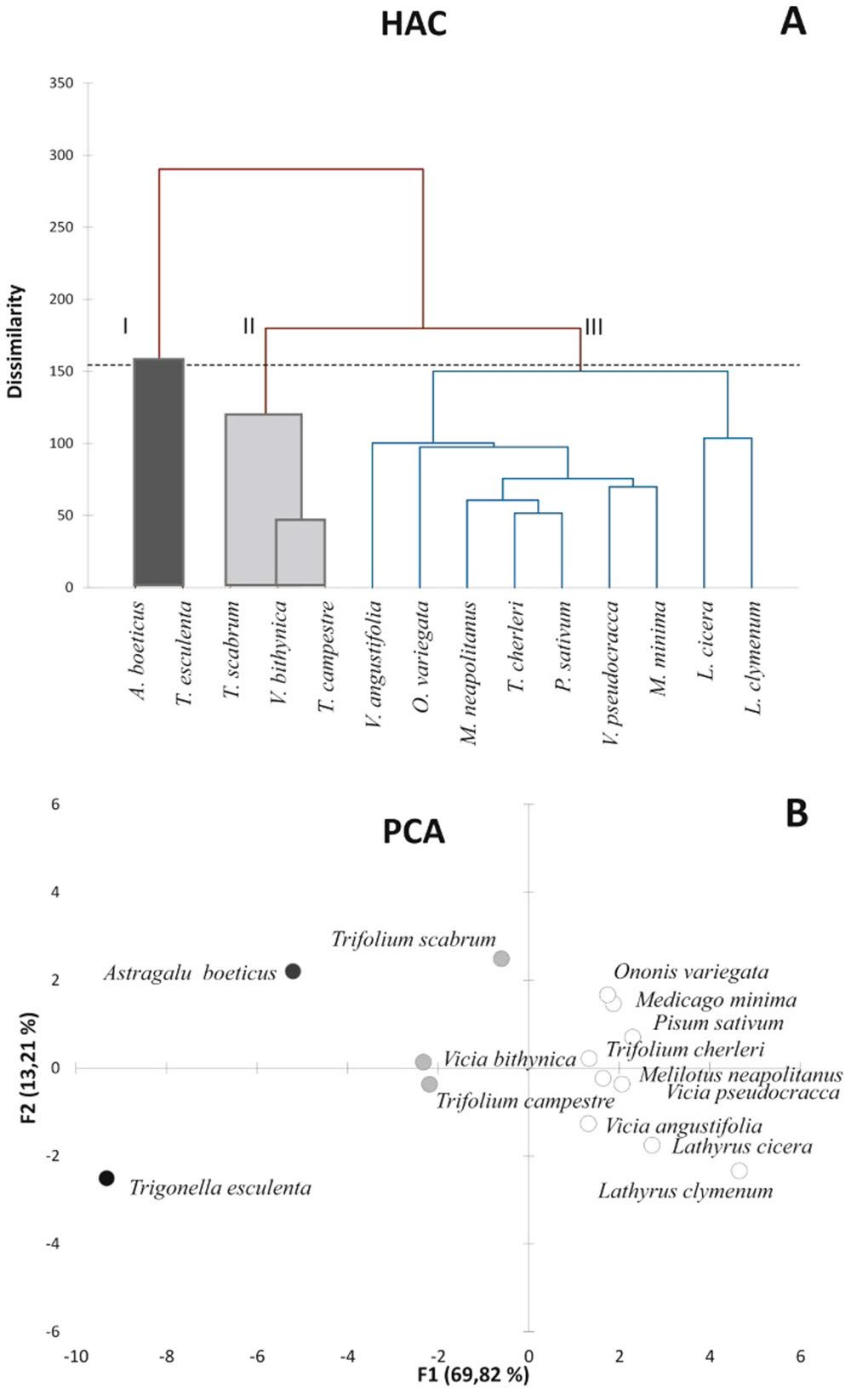
HCA dendrogram (**A**) and PCA (**B**) of cell growth percentage from control of colon cancer cell lines treated with the selected plant extracts over 48 hours. Based on these analyses the species are classified in three subsets. Groups I included the active species (black), II those active only at the highest tested doses (grey) and III those that have no-significant effect (white).

Cancer cells were treated with increasing doses of plant extracts ranging between 10 to 250 µg/ml for 48 hours and their potential cytotoxicity was assessed trough MTT assays. The resulting data expressed as percentage values of the cell growth respect to the control were used to develop multivariate statistical analyses, in particular the agglomerative hierarchical clustering (HCA) and the principal component analysis (PCA), which can provide an overview of the results as well as a categorisation of the experimental dataset. Indeed, the HCA dendrogram clearly pointed out three different clusters with a high dissimilarity degree (150%). The first group included all treatments with *A. boeticus* and *T. esculenta* plant extracts, the second was formed by *T. scabrum*, *V. bithynica* and *T. campestre*, while the third cluster encompassed all of the remaining plants (Fig. 1A). This trend was further confirmed by PCA (Fig. 1B), where in the score scatter plot the three plant groups were mainly arranged along a gradient generated by the first component, which accounted for 72.84% of the variance. This, therefore, indicated an extreme diverse anti-proliferative activity amongst the investigated plant extracts pointing out that the maximum effect on the cell growth was caused by the above-mentioned first group. In particular, *T. esculenta* showed a similar strong anti-proliferative effect on the Caco-2, HT-29 and HCT-116 cell lines, whilst *A. boeticus* displayed a mild activity on Caco-2 and HCT-116 cell lines but a selective and notable anti-proliferative effect on HT-29 cell line. These findings suggested that *A. boeticus* extract could operate with a selective mode of action on the HT-29 colon cancer cells.

**Figure 2:**
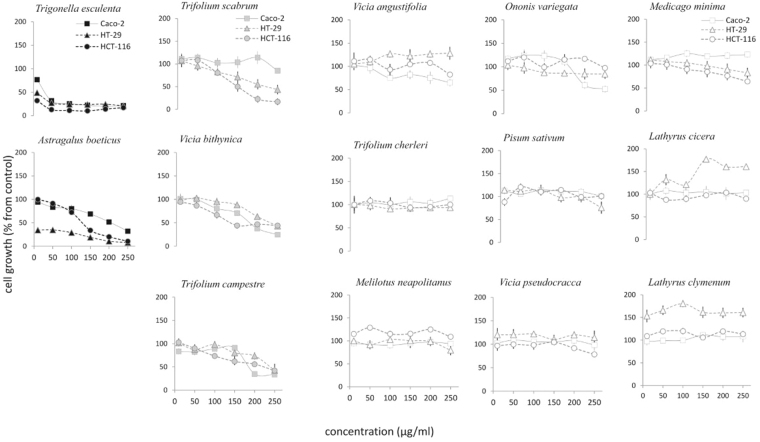
Cytotoxicity of the studied Fabaceae species on Caco-2, HT-29 and HCT-116 colorectal cancer cell lines evaluated by MTT assays over 48 hours. Cell growth is expressed as percentage from control and it is plotted in the vertical scale, while doses of plant extracts are depicted in the horizontal axis. Groups I, II and III are depicted in black; grey and white, respectively.

The second group identified in the PCA included plants that showed a severe anti-proliferative effect only at the highest tested concentrations (200 and 250 μg/ml). The rest of the screened plants (the third group) did not exhibit cytotoxic activity under the experimental conditions used and in some cases (*Latirus* spp vs. HT-29 cell lines) they exerted a stimulating effect on the cell growth. Based on these results, an extensive 2D NMR analysis was performed on the active plant extracts (*A. boeticus* and *T. esculenta*), with the aim of identifying the compounds in the mixtures that were responsible for the observed cytotoxicity (Fig. 2).

### 2D NMR analysis of the active plant extracts

#### Astragalus boeticus

**Figure 3:**
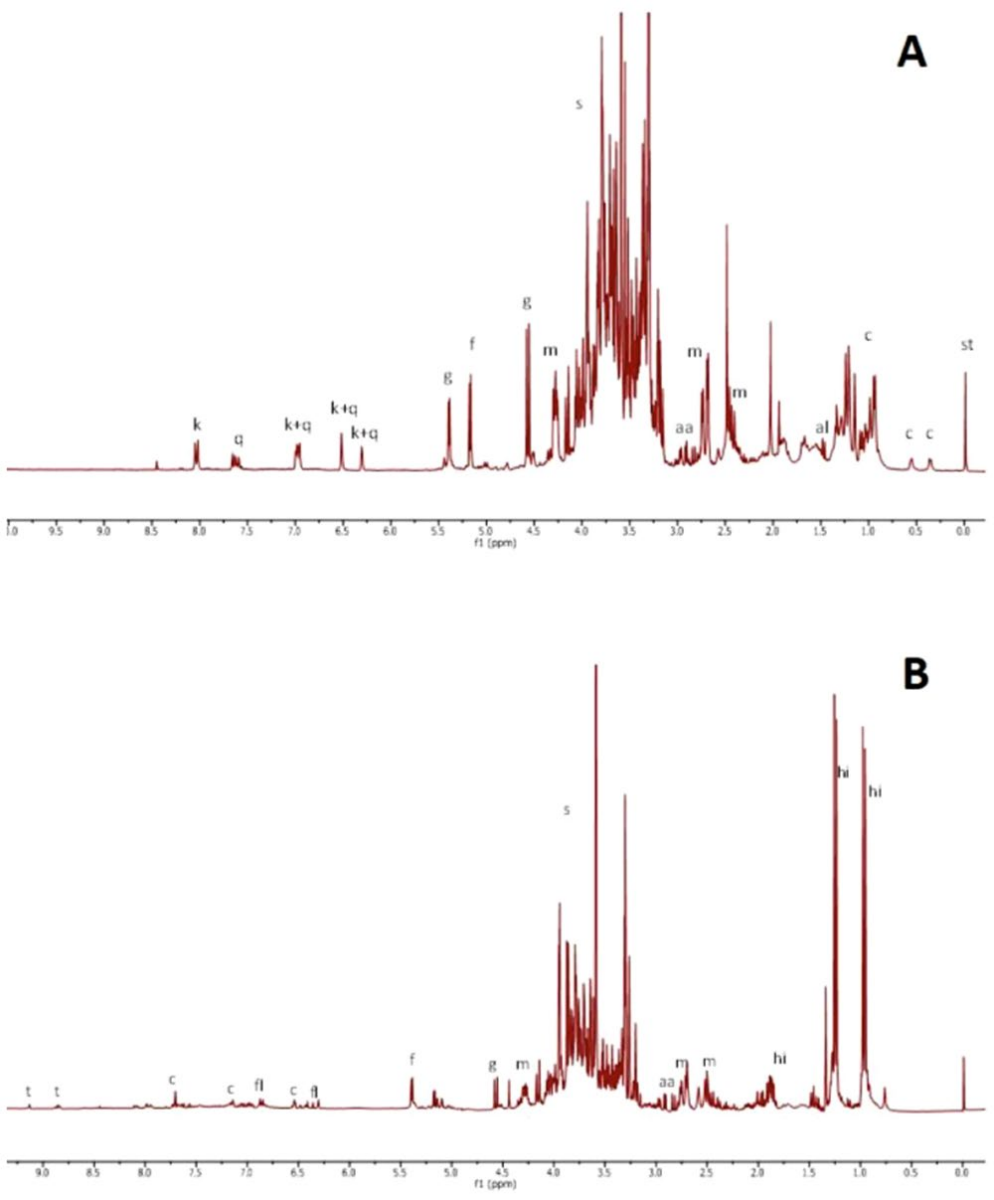
(**A**) ^1^H NMR of *Astragalus boeticus* extract registered in phosphate buffer in D_2_O (pH 6.0) and methanol d_4_ (1:1). (**B**). ^1^H NMR of *Trigonella esculenta* registered in phosphate buffer in D_2_O (pH 6.0) and methanol d_4_ (1:1). ABBREVIATIONS: aa, aspartic acid; al, alanine; c, cycloartane; fl, flavonoids; f, fructose; g, glucose; h, 4- hydroxyisoleucine; k, kaempferol; malic acid; q, quercetin; st, standard; s, sugar; t, trigonelline.

**Figure 4:**
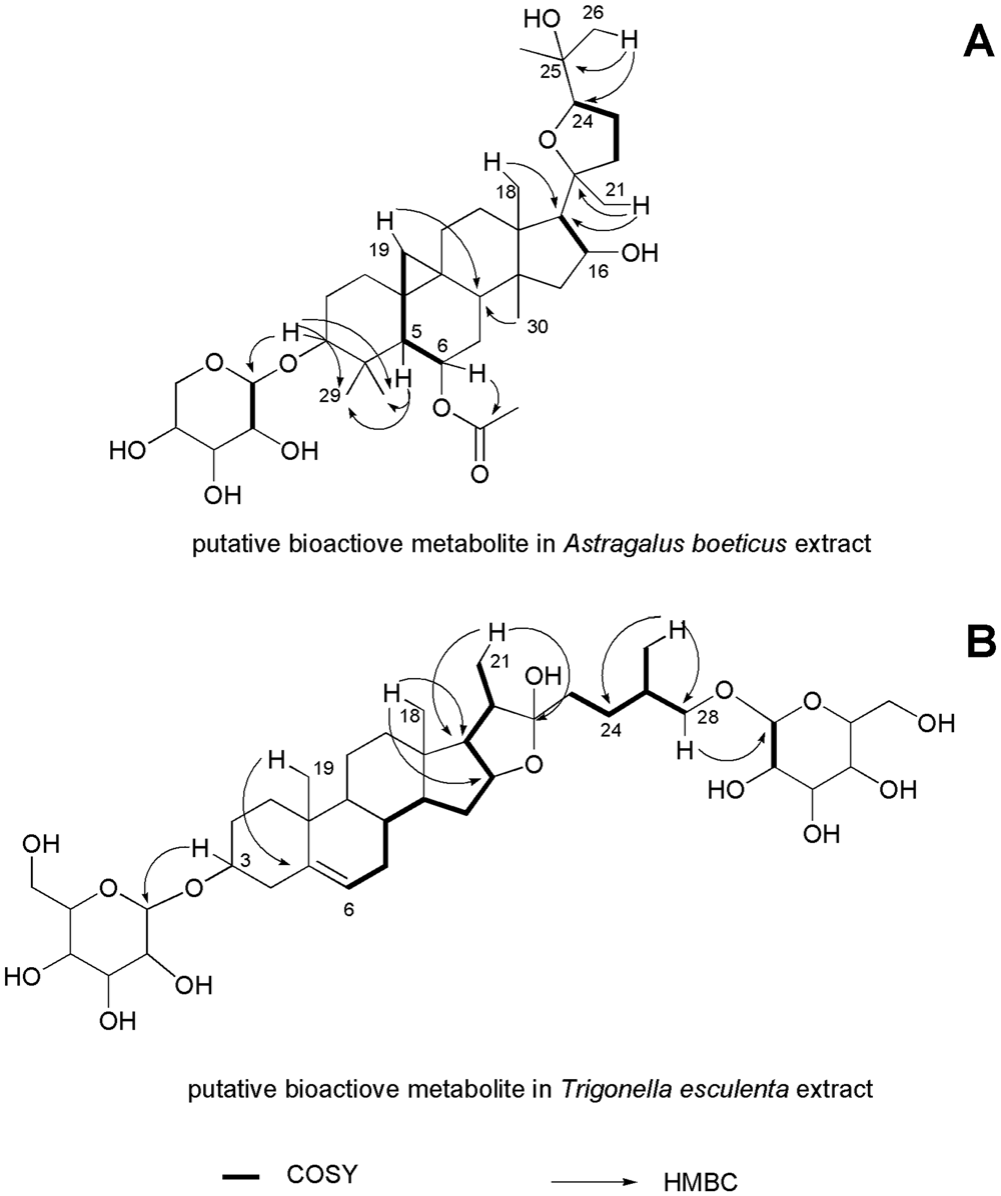
Main COSY and HMBC correlations of the putative bioactive metabolites in *Astragalus boeticus* and *Trigonella esculenta* extracts.

The NMR analysis of *A. boeticus* extract showed the presence of three main secondary metabolites. The first two molecules were identified as quercetin and kaempferol (Table S1), two flavonols widely distributed in the plant kingdom, while the third metabolite was a cycloartane triterpene. Beyond the methylene H-19 protons at δ_H_ 0.37 and 0.57, seven methyl singlets were clearly detectable in the ^1^H NMR spectrum (Fig. 3A). In detail, signals at δ_H_ 1.01 (δ_C_ 15.5) and 1.06 (δ_C_ 25.7) were assigned to the H-28 and H-29 methyls, based on HMBC heterocorrelations (Fig. 4A) with C-5 methine (δ_C_ 49.5), carbon that was found to bind the proton at δ_H_ 1.66. This proton (H-5) correlated with the H-19 methylene and H-6 proton in the COSY experiment; this resonated at δ_H_ 4.75 suggesting the presence of an oxygen atom binding the C-6 carbon. Furthermore, both C-28 and C-29 carbons correlated with the H-5 methine and H-3 proton at δ_H_ 4.44 (δ_C_ 88.4). Besides, the H-19 protons displayed heterocorrelations with the C-8 methine carbon (δ_C_ 45.0), which in turn correlates with the H-30 methyl that resonated at δ_H_ 0.99 (δ_C_ 19.2). Furthermore, the signal at δ_H_ 1.24 (δ_C_ 20.3), attributed to the 18 methyl, and the peak at δ_H_ 1.26 (δ_C_ 27.2), related to the H-21 methyl, correlated with the C-17 carbon at δ_C_ 57.7. In the COSY spectrum, the proton bound to this carbon revealed homocorrelations with the H-16 proton at δ_H_ 4.64; this chemical shift value was well in agreement with the presence of an hydroxyl group in this position. A series of HMBC correlations, which were found between the H-21 proton and the C-20 carbon (δ_C_ 87.6); the H-26 methyl (δ_H_ 1.24) and the C-25 carbinol (δ_C_ 72.0); the H-27 methyl (δ_H_ 1.34) and the C-24 carbinol (δ_C_ 81.2) allowed us to hypothesise the presence of a substituted tetrahydrofuran moiety in the side chain of the triterpene. All these data were in agreement with the presence of a cycloastragenol^25,26^. The downfield shift of the H-3 and C-3 values justified the presence of a sugar moiety, indicating a presumable site of glycosylation at position 3. This hypothesis was validated with an HMBC experiment, in which the C-3 carbon clearly heterocorrelated with the anomeric proton at δ_H_ 4.44 (δ_C_ 107.0). Finally, the H-6 proton showed cross peak with a carbonyl at δ_C_ 172.5, which in turn correlated with the methyl at δ_H_ 2.04 (δ_C_ 21.2). These data demonstrated the presence of an acetyl group, which formed an ester bond with the hydroxyl group located at C-6 carbon.

#### Trigonella esculenta

*T. esculenta* metabolome presented little variability in terms of secondary metabolites; indeed, the ^1^H spectrum was dominated by of 4-hydroxyisoleucine signals (Fig. 3B). In an attempt to see beyond these primary metabolites, the crude extract was purified on a Sep-pak C-18 cartridge and the resulting methanol eluate was analysed by NMR. In the up-field region of the spectrum, peculiar doublets and singlets between 4.2 and 5.2 ppm suggested the presence of triterpenoid saponins. In particular, two singlets at δ_H_ 0.83 and 1.04, alongside two doublets at δ_H_ 0.94 and 1.01 suggested the presence of a steroid moiety^27^. In the HMBC experiment (Fig. 4B), the doublet at δ_H_ 0.94 (δ_C_ 17.6) correlated either with the methine C-24 at δ_C_ 35.2 or a methylene carbinol C-28 at δ_C_ 75.9 (δ_H_ 3.70 and 3.40). This signal showed cross peaks with the doublet at δ_H_ 4.73 (δ_C_ 104.8), indicating a glycosylation site at position 28. Moreover, the doublet at δ_H_ 1.01 (δ_C_ 15.7) heterocorrelated with two methines at δ_C_ 40.8 and 64.6, and with the acetal carbon at δ_C_ 113.9. These data are consistent with the presence of a furostanol moiety in the molecule. A second site of glycosylation was identified at position 3; indeed, the carbinol carbon at δ_C_ 79.2 correlated with the anomeric proton at δ_H_ 4.40. Moreover, overlapped doublets between 1.20 and 1.30 ppm correlated in the HSQC experiment with carbons at δ_C_ 18.4, 18.1 and 17.9. Finally, the correlations of these protons with carbinol carbons supported the presence of three deoxy sugars.

### Isolation and identification of the active metabolites from the cytotoxic plant extracts

#### Compound A from Astragalus boeticus

**Figure 5:**
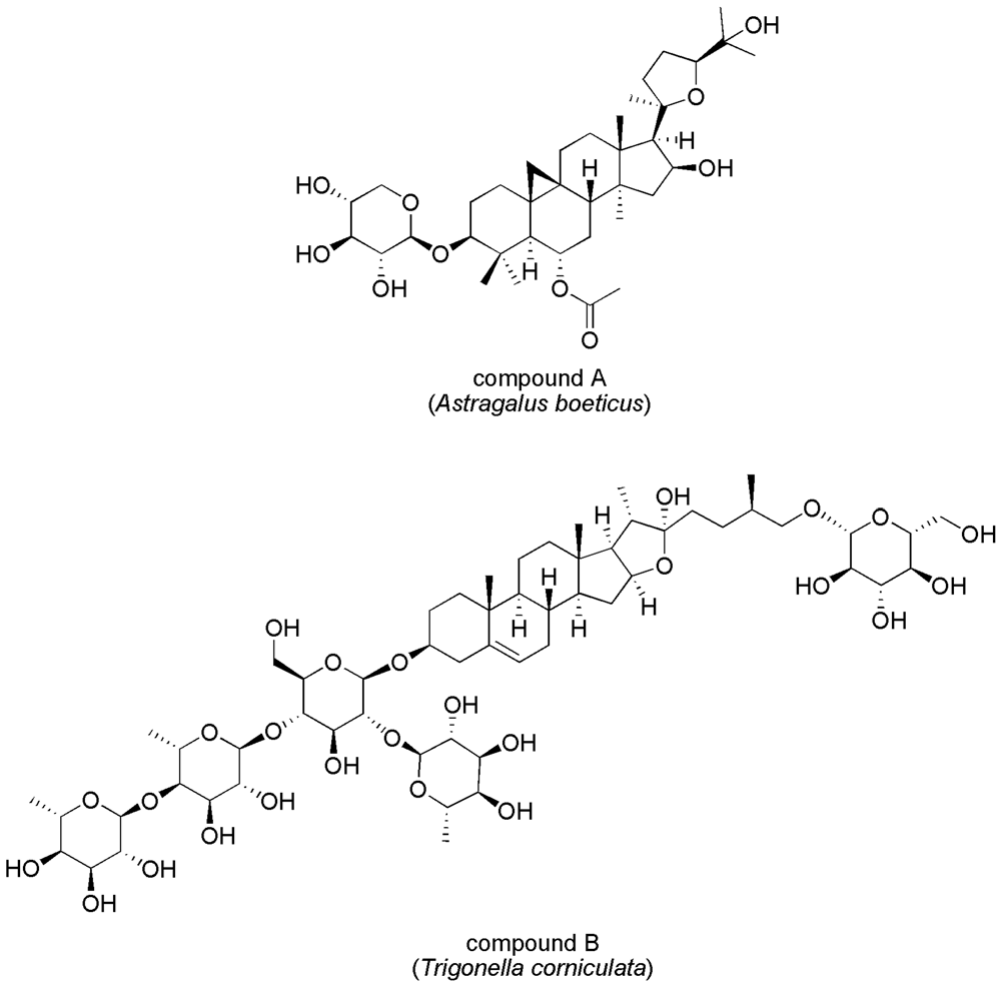
Bioactive constituents of *Astragalus boeticus* (compound A) and *Trigonella esculenta* (compound B) extracts.

*A. boeticus* crude extract was partitioned between ethyl acetate and water; after purification through different chromatographic processes, the organic phase led to the isolation of the pure compound A^28^ (Fig. 5). It showed a molecular formula C_37_H_60_O_10,_ according to the ^13^C NMR data and the positive ESI Q-TOF HRMS spectrum that displayed a sodiated adduct at *m/z* 687.4480. In the ^1^H NMR spectrum, characteristic signals of a cycloartane triterpene were detected: two methines at δ_H_ 0.61 and 0.40 (H-19) and seven singlet methyls at δ_H_ 1.27, 1.26, 1.22, 1.13, 1.09, 1.01, 0.99. The presence of a doublet at δ_H_ 4.26, as well as other protons resonating between 3.18 and 3.46 ppm, suggested the presence of sugar in the molecule. The 2D NMR experiments (COSY, TOCSY, NOESY, HSQC, CIGAR-HMBC, H2BC and HSQCTOCSY) were in agreement with the presence of a cycloatragenol as aglycone. In particular, in the CIGAR-HMBC experiment the H-6 proton at δ_H_ 4.75 showed a cross peak with the ester carbonyl at δ_C_ 171.7, which in turn correlated with the methyl at δ_C_ 1.99. These data suggested the presence of an acetate group at position 6. Moreover, a glycosylation site at position 3 was supported by the correlation between H-3 methine at δ_H_ 3.21 and the anomeric carbon at δ_C_ 107.4. The TOCSY, HSQCTOCSY and H2BC NMR experiments allowed the identification of the saccharide moiety as xylose, while the coupling constant value of the anomeric proton (6.0 Hz) agreed with a β configuration. Finally, the stereochemistry of compound A was defined using a NOESY experiment. Taken all together, these data enabled the identification of compound A as 6-O-acetyl-3-O-β-D xylopiranosylcycloastragenol. This was confirmed by tandem MS analysis of the pseudomolecular ion [M + Na]^+^ that showed the presence of the fragment ion at *m/z* 537.3399 and 627.3801, due to the loss of the xylose and the acetate, respectively.

#### Compound B from Trigonella esculenta

The crude extract of *T. esculenta* was chromatographed on Amberlite XAD-4 and eluted with water to eliminate polar metabolites. The saponin fraction, recovered by methanol, was fractionated through column chromatography and finally purified by RP-HPLC to obtain pure compound B^29,30^ (Fig. 5). It showed a molecular formula C_57_H_94_O_26_, according to the positive ESI Q-TOF HRMS analysis and ^13^C NMR data. The ^1^H NMR spectrum showed the signals of the steroidal aglycone as two singlet methyls at δ_H_ 0.84 and 1.05, and two doublets at δ_H_ 0.95 and 1.01.

In the ^1^H NMR spectrum, different anomeric protons suggested the presence of an oligosaccharide, which was located at C-3 carbon, thanks to the CIGAR-HMBC experiment. The HSQCTOCSY and H2BC experiment in combination with the Q-TOF HRMS analysis further confirmed this structure. Thus, compound B was unequivocally identified as [(25 R)-furost-5-ene-3β,22α,26-triol 3-O-α-L-rhamnopyranosyl-(1 → 4)-α-L-rhamnopyranosyl-(1 → 4)-[α-L-rhamnopyranosyl-(1 → 2)]-β-D-glucopyranosyl 26-O-β-D-glucopyranoside.

### Anti-proliferative activity of pure compounds

In an attempt to understand whether the isolated compounds were actually responsible for the cytotoxicity of the crude extracts, their cytotoxicity was evaluated against a panel of colorectal cancer cell lines (Caco-2, HT-29, HCT-116). In these assays, a wide range of treatment concentrations was examined over 48 hours.

With regard to *A. boeticus*, after incubating the cells with compound A at concentrations below 50 µM, a reduction of approximately 50% of cell growth was observed (Table 1). This strong antiproliferative effect resembled closely what had been observed with the crude plant extract. Interestingly, this metabolite did not exert the same anti-proliferative effect on the three types of colon cancer cells tested, as its IC_50_ value was significantly lower for the HT-29 cells (IC_50_ = 3 µM). This may suggest a selective mode of action of compound A on this particular cell type.

**Table 1:** IC_50_ values of compound A and B evaluated by MTT assays over 48 hours on Caco-2, HT-29, HCT-116 colorectal cancer cell lines.

Active metabolites	Caco-2	HT-29	HCT-116
Compound A	50 µM	3 µM	40 µM
Compound B	3 µM	3 µM	2 µM

With regard to *T. esculenta*, compound B was able to recapitulate the cytotoxic activity of the crude plant extract (Table 1). After the incubation of Caco-2, HT-29 and HCT-116 cell lines, a strong anti-proliferative effect was detected and very low doses (<10 µM) reduced cell growth by approximately 50%, indicating that this metabolite was largely accountable for the anti-proliferative effect of the plant extract. Compound B displayed the same effect on Caco-2 and HT-29 (IC_50_ = 3 µM), while its activity was slightly higher in HCT-116 cell lines (IC_50_ = 2 µM).

To further validate that A and B were the active constituents of *A. boeticus* and *T. esculenta*, the other main secondary metabolites, which were previously identified in these plant derived-mixtures, were isolated and tested. In detail, we purified the flavonols quercitin and kaempferol from the first plant extract, and the amino acid 4-hydroxyisoleucine from the second. We subsequently evaluated their cytotoxicity on the selected human colon cancer cell lines, demonstrating that these have no significant effect under the examined conditions.

## Discussion

Natural product research requires new strategies to renovate the traditional methodologies, which are too expensive and/or time consuming^31,32^. Metabolomics ameliorate the effectiveness of previous approaches restoring the relevance of natural products as source of novel potential anticancer agents^33,34^. Here, we integrate metabolomic strategies with biological assays to search secondary metabolites from Fabaceae species, which exert cytotoxicity against colon cancer cells.

We performed a detailed metabolomic analysis of fourteen Fabaceae species, unambiguously identifying 31 metabolites from these plants. With regard to the non-active species, there is a fragmented literature that described these from different points of view^24,35–38^. Nonetheless, the current research unveiled a more comprehensive picture of the studied species, showing their metabolomic profiles and pointing out the main secondary metabolites present in the mixture. Because of their strong cytotoxic effect on Caco-2, HT-29 and HCT-116 human colorectal cancer cell lines, *A. boeticus* and *T. esculenta* extracts were selected for further investigations. To our knowledge, there is a little available data about these selected plant extracts; however, previous published work on other species belonging to the same genus assisted us in the search and identification of the putative active constituents of our selected plants.

In Traditional Chinese Medicine (TCM) *Astragalus* genus is particularly widespread for its antiperspirant, diuretic, and tonic properties. In particular *A. membranaceus*, is very well-known in folk medicine for its anti-inflammatory and immunomodulatory effects mainly exerted by regulating the (NF)-κB signalling pathways^39–41^. Furthermore, recent studies have proven that cycloartane and oleanan-type saponins modulate the cytokine release and might contribute either to the anti-inflammatory or anti-proliferative effect of *Astragalus* spp.^42^. It has been also demonstrated that *Astragalus* saponins from *A. membranaceus* regulate cell invasiveness and angiogenesis in human gastric adenocarcinoma cells^43^.

*Trigonella esculenta* has been already investigated for its ethyl-galactoside^44^ and diosgenin contents^45^, yet no previous studies evaluated the anti-proliferative activity of this species. The majority of literature data refers to *Trigonella foenum-graecum L*. (Fenugreek), a medicinal and dietary plant to which were attributed a very wide range of biological properties^46^, especially those regarding to the hypocholesterolaemic and hypoglycaemic effect^47,48^. Notably, other previous works reported the potentiality of *T. foenum-graecum* as source of potential anti-cancer agents: in detail, anti-proliferative metabolites have been identified to be steroidal furostan-type saponins, such as diosgenin and protodioscin^49,50^.

In this work, we sought to identify the compounds responsible for plant extract cytotoxicity against colorectal cancer cells, and to this aim an extensive 2D NMR analysis of the active crude extracts was carried out followed by a bio-guided fractionation to isolate these metabolites. Consistently with the previous investigations, we found a cycloartane glycoside (compound A) and a protodioscin derivative (compound B) as active principles from *A. boeticus* and *T. esculenta*, respectively (Fig. 5).

A previous work described compound A and its capacity to inhibit nitric oxide production, identifying this metabolite as a potential anti-inflammatory agent^27^. Nevertheless, here the proliferation reducing activity of compound A was demonstrated for the first time. Supporting our findings, antineoplastic properties have already been associated with other cycloartane glycosides. Of these, astragaloside I – II – III isolated from *A. membranaceus* have been tested against a panel of different human cancer cells, which represent diverse common malignancies. The strongest anti-proliferative and apoptotic effect was observed in the leukemic (HL-60, HL-60/Dox and SKW-3) and in breast cancer (MDA-MB-231) cell lines, while the colorectal carcinoma НТ-29 was found to be the least responsive to the saponin treatment^51^. This latter finding is not in full agreement with our results, where compound A was found to be most active in HT-29 cells (IC50 = 3 µM) compared with the other studied colon cancer cell lines, Caco-2 (IC50 = 50 µM) and HCT-116 (IC50 = 40 µM). Another study described astragaloside II as a novel promising agent for reversal of multidrug resistance (MDR) mediated by P-glycoprotein in human hepatic cancer cells^52^.

Our work established for the first time the presence of compound B in *Trigonella spp*., albeit this metabolite has already been isolated from other genus. Previous evidence proved that fractions deriving from *Livistona chinensis* var *subglobosa* seeds containing compound B promote apoptosis in different human cancer cells and, thus, it could be used to both prevent and treat cancer (US 2010/0179098 A1). Another invention patent included this molecule in a pharmacological composition together with a histone deacetylase inhibitor, the combination of these compounds causes an increase of the apoptotic rate (US 2012/0058961 A1). Here, we found that compound B has a potent anti-proliferative effect towards the investigated panel of colon cancer cell lines, with a slight preferentiality for HCT-116.

Interestingly, compound A and B displayed an enhanced effect on HT-29 and HCT-116 cells, respectively. These cell lines are characterised by genetic defects in BRAF and KRAS/PIK3CA, respectively. KRAS mutation, or genetic variation in other intracellular downstream effectors of EGFR activation, such as BRAF, NRAS, and PIK3CA, are responsible for the intrinsic resistance to anti-EGFR therapies^53,54^. Moreover, the remarkable selectivity of compound A for HT-29 may has a potent clinical impact; since BRAF mutated mCRC patients have a very negative prognosis. In fact, for this pathology at present there is neither a standard chemotherapeutic treatment nor targeted therapies available. Although inhibition of BRAF oncoprotein by the small-molecule drug PLX4032 (vemurafenib) is highly effective in the treatment of melanoma, colon cancer patients associated with the same BRAF mutation have poor prognosis showing only a very limited response to this drug. As Vemurafenib treatment induces EGFR feedback activation, this may explain the refractoriness of BRAF (V600E) mutated colon cancers to this therapy^55^.

In conclusion, using an innovative and highly efficient screening approach we quickly identified two natural products (A and B) as potent cytotoxic molecules against human colorectal cancer cells, which exert resistance against anti-EGFR therapies. These findings encouraged further investigations to understand the mode of action of compounds A and B and their potential application in drug resistant colon cancer therapy. Obviously, different limitations are intrinsically associated to our *in vitro* experimental system; and for this reason, future experiments will be also addressed to evaluate the pharmacokinetic properties and the bioavailability of the active metabolites in animal models.

## Materials and Methods

### Plant material

Plant species were harvested in April 2014 at the “Castel Volturno” Nature Reserve (40°57.587′N, 14°00.105′E; southern Italy), identified and the voucher specimens were deposited at the Herbarium of DiSTABiF of Università degli Studi della Campania “Luigi Vanvitelli, as reported below: *A. boeticus* L, *Lathyrus cicera* L., *Lathyrus clymenum* L., *Medicago minima* (L.) L., *Melilotus neapolitanus* Ten., *Ononis variegata* L., *Pisum sativum* L. subsp. *biflorum* (Raf.) Soldano, *Trifolium campestre* Schreb., *Trifolium cherleri* L., *Trifolium scabrum* L. subsp. *scabrum*, *Trigonella esculenta* Willd., *Vicia bhytinica* (L.) L., *Vicia pseudocracca* Bertol., *Vicia angustifolia* L.. Leaf samples were collected and immediately frozen in liquid N_2_ to avoid unwanted enzymatic reactions and stored at −80 °C before the freeze-drying process. Lyophilized samples were powdered in liquid nitrogen and stored at −20 °C.

### Extraction procedure for metabolomic analysis

An aliquot (50 mg) of freeze-dried and powdered plant material was transferred to a 2 mL microtube. 1.5 mL of phosphate buffer (Fluka Chemika, Buchs, Switzerland; 90 mM; pH 6.0) in D_2_O (Cambridge Isotope Laboratories, Andover, MA,USA) - containing 0.1% w/w trimethylsilylpropionic-2,2,3,3-d_4_ acid sodium salt (TMSP, Sigma–Aldrich, St. Louis, MO, USA)- and CD_3_OD (Sigma–Aldrich, St. Louis, MO, USA) (1:1) were added to the samples. The mixture was vortexed at room temperature for 1 min, ultrasonicated (Elma Transsonic Digital, Hohentwiel, Germanys) for 40 min, and centrifuged (Beckman Allegra™ 64 R, F2402H rotor; Beckman Coulter, Fullerton,CA, USA) at 13,000 rpm for 10 min. A volume of 0.65 mL was transferred to a 5-mm NMR tube and analyzed by NMR^56^.

### NMR experiments

NMR spectra were recorded at 25 °C on a 300.03 MHz for ^1^H and 75.45 MHz for ^13^C on a Varian Mercury Plus 300 Fourier transform NMR. CD_3_OD was used as the internal lock. Each ^1^H NMR spectrum consisted of 256 scans with the following parameters: 0.16 Hz/point, acquisition time (AQ) = 1.0 s, relaxation delay (RD) = 1.5 s, 90° pulse width (PW) = 13.8 μs. A presaturation sequence was used to suppress the residual H_2_O signal. FIDs were Fourier transformed with LB = 0.3 Hz. The spectra were manually phased and baseline-corrected and calibrated to TMSP at 0.0 ppm.

High-resolution experiments were performed, in particular 2D-NMR spectra were used for extracts *A. boeticus* and *T. esculenta* extracts to identify the putative cytotoxic metabolites. Standard pulse sequences and phase cycling from Varian library were used for ^1^H, DQF-COSY, COSY, TOCSY, HSQC, H2BC, HSQCTOCSY, HMBC and CIGAR-HMBC experiments. COSY spectra were acquired with a 1.0 s relaxation delay and 2514 Hz spectral width in both dimensions. The window function for COSY spectra was sine-bell (SSB = 0). Proton-detected heteronuclear correlations were measured. Heteronuclear single-quantum coherence (HSQC) experiments (optimized for ^1^J_(H,C)_ = 140 Hz) were performed in the phase sensitive mode with field gradient. The spectral width was 12000 Hz in f1 (^13^C) and 3000 Hz in f2 (^1^H) and 1.0 s of relaxation delay; the matrix of 1 k × 1 k data points was zero-filled to give a final matrix of 2 k × 2 k points. Heteronuclear 2 bond correlation (H2BC) spectra were obtained with T = 30.0 ms, and a relaxation delay of 1.0 s; the third-order low-pass filter was set for 130 < ^1^J_(C,H)_ < 165 Hz. Heteronuclear multiple bond coherence (HMBC) experiment (optimized for ^n^J_(H,C)_ = 8 Hz) was performed in the absolute value mode with field gradient. Typically, ^1^H–^13^C gHMBC were acquired with spectral width of 18116 Hz in f1 (^13^C) and 3140 Hz in f2 (^1^H) and 1.0 s of relaxation delay; the matrix of 1 k × 1 k data points was zero-filled to give a final matrix of 4 k × 4 k points; Qsine (SSB = 2.0). Constant time inverse-detection gradient accordion rescaled heteronuclear multiple bond correlation spectroscopy (CIGAR–HMBC) spectra (8 > ^n^J_(H,C)_ > 5) were acquired with the same spectral width used for HMBC. Heteronuclear single-quantum coherence - total correlation spectroscopy (HSQC-TOCSY) experiments were optimized for ^n^J_(H,C)_ = 8 Hz, with a mixing time of 90 ms. Free induction decays (FIDs) were Fourier transformed and the resulting spectra were manually phased and baseline-corrected and calibrated to TMSP at 0.0 ppm, using ^1^H NMR processor (ACDLABS 12.0, Toronto, Canada). For accurate mass measurements the purified compounds were analyzed by electrospray hybrid quadrupole orthogonal acceleration time-of-flight mass spectrometer (Q-TOF) fitted with a Z-spray electrospray ion source (Waters S.p.A.). All analyses were performed in both positive and negative ion mode. The capillary source voltage and the cone voltage were set at 3500 V and 35 V, respectively. The source temperature was kept at 80 °C and nitrogen was used as a drying gas (flow rate about 50 L/h). The time-of-flight analyzer of the mass spectrometer was externally calibrated with GFP from *m/z* 50 to 1600. Accurate mass data were collected by directly infusing samples (1.5 pmol/μL in CH_3_CN/H_2_O, 1:1) into the system at a flow rate of 15 μL/min. The acquisition and processing of data were performed with the MassLynx 4.1 software (Waters S.p.A., Manchester, UK).

### Isolation of active metabolites

#### Extraction, isolation and identification of compound A from *Astragalus boeticus*

Dried leaves (24.0 g) of *A. boeticus* were powdered and underwent to three cycles of an ultrasound assisted extraction with a MeOH/H_2_O (1:1) solution (720 mL), obtaining a crude extract (7.1 g). This was dissolved in H_2_O and separated by liquid–liquid extraction using EtOAc as an extracting solvent. The EtOAc fraction (1 g) was chromatographed by SiO2 CC eluting with a solution with an increasing degree of polarity (CHCl_3_, Me_2_CO/ CHCl_3_, MeOH/ CHCl_3_). 21 fractions have been collected, number 12 was fractionated by Flash-CC eluting with MeOH/ CHCl_3_ (3:100) to obtain compound A (28 mg). Compound A (6-O-acetyl-3-O-β-d xylopiranosylcycloastragenol). [α]D^25^ =  + 7,65 (c = 5,1 × 10–3, MeOH/H2O, 2:1).^13^C-NMR (CD_3_OD): see Table S1; ^1^H-NMR (CD_3_OD):see Table S1. Q-TOF HRMS (ESI^+^): *m/z* 703.3877 [M + K]^+^ (calcd. 703.3818 for C_37_H_60_O_10_K^+^), 687.4080 [M + Na]^+^ (calcd. 687.4079 for C_37_H_60_O_10_Na^+^), 627.3801 [M-AcOH + Na]^+^ (calcd. 627.3873 for C_35_H_56_O_8_Na^+^), 537.3399 [M-C_5_H_10_O_5_ + Na]^+^ (calcd. 537.3556 C_32_H_50_O_5_Na^+^), 477.3164 [M-AcOH-C_5_H_10_O_5_ + Na]^+^ (calcd. 477.3345 C_30_H_46_O_3_Na^+^).

#### Extraction, isolation and identification of compound B form *Trigonella esculenta*

Dried leaves (10.0 g) of *T. esculenta* Willd were powdered and underwent to three cycles of an ultrasound assisted extraction with a MeOH/H_2_O (1:1) solution (300 ml) obtaining a crude extract (5.0 g), which was dissolved in H_2_O and separated by liquid–liquid extraction using EtOAc as an extracting solvent. The aqueous fraction was chromatographed on Amberlite XAD-4 and XAD-7 with water and then with methanol. The alcoholic eluate from both XAD-4 and XAD-7 (1.2 g) was chromatographed by Sephadex LH-20 CC eluting with hydroalcoholic solution (MeOH/H_2_O) with an increasing percentage of methanol to collect 8 fractions (20 mL each). Fractions 2 and 3 have been purified by Flash CC eluting with the lower phase of CHCl_3_/MeOH/H_2_O (13:7:3) solution to give another fraction (74.0 mg), which in turn was chromatographed through HYDRO-RP 80 HPLC eluting with H_2_O/MeOH/MeCN (2:2:1) providing pure compound B (52.0). Compound B ([(25 R)-furost-5-ene-3β,22α,26-triol 3-O-α-L-rhamnopyranosyl-(1 → 4)-α-L-rhamnopyranosyl-(1 → 4)-[α-L-rhamnopyranosyl-(1 → 2)]-β-D-glucopyranosyl 26-O-β-D-glucopyranoside. [α]_D_^25^ =  + 5,5 (c = 5,1 × 10^-3^, MeOH/H_2_O, 2:1).^13^C-NMR (CD_3_OD): S2; ^1^H-NMR (CD_3_OD): see Table S1;

Q-TOF HRMS (ESI^+^): *m/z* 1217.5895 [M + Na]^+^ (calcd. 1217.5926 for C_57_H_94_O_26_Na^+^), *m/z* 1177.6051 [M-OH]^+^ (calcd. 1177.6006 for C_57_H_93_O_25_^+^); TANDEM MS of 1177.6051: 1015.5361 [M-OH-Glc]^+^ (calcd. 1015.5478 for C_51_H_83_O_20_), 869.4803 [M-OH-Glc-Rha]^+^ (calcd. 869.4899 for C_45_H_73_O_16_), 723.4020 [M-OH-Glc-2Rha]^+^ (calcd. 723.4319 for C_39_H_63_O_12_), 577.3481 [M-OH-Glc-3Rha]^+^ (calcd. 577.3740 for C_33_H_53_O_7_), 415.3236 [M-OH-2Glc-3Rha]^+^ (calcd. 415.3212 for C_27_H_43_O_3_).

### Cell Lines

The human HCT-116, HT-29, Caco-2 colorectal cancer cell lines were obtained from the American Type Culture Collection (ATCC) (Manassas, VA). HCT-116, HT-29 cancer cells were cultured in RPMI 1640 medium (Lonza, Cologne, Germany) supplemented with 10% fetal bovine serum, 2 mM L-glutamine, 50 U/ml penicillin and 100 µg/ml streptomycin (Lonza, Cologne, Germany). Caco-2 cell line was cultured in DMEM medium (Lonza, Cologne, Germany) supplemented with 10% fetal bovine serum, 2mM L-glutamine, 1% non-essential amino acid, 50 U/ml penicillin and 100 µg/ml streptomycin (Lonza, Cologne, Germany).

### Proliferation Assay

Cell proliferation assay was measured with 3-(4,5-dimethylthiazol-2-yl)-2,5-diphenyltetrazolium bromide (MTT) assay. Briefly, cells in logarithmic growth phase were plated in 96-well plates and incubated for 24 hours before exposure to increasing doses of plant extracts (10, 50, 100, 150, 200 and 250 µg/ml), enriched in secondary metabolites by a Sep-pak C18 cartridge step. 48 hours after treatment, 50 µl of 1 mg/ml (MTT) were mixed with 200 μl of medium and added to the well. 1 hour after incubation at 37 °C, the medium was removed and the purple formazan crystals produced in the viable cells were solubilized in 100 μl of dimethyl sulfoxide and quantitated by measurement of absorbance at 570 nm with a plate reader. Results were reported as mean ± sd of % of cell growth respect to the control from six replicate analyses. The control was represented by 0.25% DMSO treatment corresponding to the higher amount of DMSO used for the tests.

### Multivariate data analysis

The data matrix of cell growth percentage from control of the plant extracts treatments has been analyzed by multivariate analysis based on XLSTAT 2013 (Addinsoft, New York, NY, USA for Microsoft Office Excel 2010). Hierarchical cluster analysis (HCA), based on Euclidean distance as dissimilarity index and complete linkage as the agglomeration method, has been carried out to evaluate statistical differences between extracts. Furthermore, Principal component analysis (PCA) was also performed as ordination methods based on XLSTAT 2013.

## Electronic supplementary material

Table S1
