# Supplementary material for: Metabolomic approach for a rapid identification of natural products with cytotoxic activity against human colorectal cancer cells
Source: Sci Rep. 2018 Mar 28;8:5309. doi: 10.1038/s41598-018-23704-9 (PMC5871890; doi:10.1038/s41598-018-23704-9)
Supplement: Supplementary file 1 — Table S1 [file 41598_2018_23704_MOESM1_ESM.pdf]

Metabolomic approach for a rapid identification of natural products with cytotoxic activity against human colorectal cancer cells

Vittoria Graziani[1,§], Monica Scognamiglio[1,2,§], Valentina Belli[3], Assunta Esposito[1], Brigida D'Abrosca[1], Angela Chambery[1], Rosita Russo[1], Marta Panella[1], Aniello Russo[1], Fortunato Ciardiello[3], Teresa Troiani[3,*], Nicoletta Potenza[1,*], Antonio Fiorentino[1,*]

[1]Dipartimento di Scienze e Tecnologie Ambientali Biologiche e Farmaceutiche (DiSTABiF), Università degli Studi della Campania "Luigi Vanvitelli", via Vivaldi 43 I-81100, Caserta, Italy
[2]Max Planck Institute for Chemical Ecology - Beutenberg Campus, Hans-Knöll-Straße 8 D-07745 Jena, Germany
[3]Dipartimento Medico-Chirurgico di Internistica Clinica e Sperimentale "F. Magrassi - A. Lanzara", Università degli Studi della Campania "Luigi Vanvitelli" I-80131 Via Pansini, 5 - Napoli, Italy

SUPPLEMENTARY INFORMATION

Table S1 Main metabolites detected in the studied plant extracts. ^{1}H-NMR data are measured in ppm and coupling constants (J) in Hertz.

Plant Species	Metabolites	Chemical shift values
Astragalus boeticus	Cycloartanes	0.37 (H19, d, J=4.6); 0.57 (H19, d, J=4.6); 0.99 (H30, s); 1.01 (H28, s); 1.06 (H29, s); 1.24 (H18, s); 1.24 (H26, s); 1.26 (H21, s); 1.34 (H27, s)
	Kaempferol	6.32 (H6, d, J = 2.1); 6.53 (H8, d, J = 2.1); 7.00 (H2'/H6', d, J = 8.4); 8.03 (H3'/H5', d, J = 8.4)
	Quercetin	6.27 (H6, d, J = 2.1); 6.52 (H8, d, J = 2.1); 6.99 (H5', d J = 8.5); 7.60 (H6', dd, J = 8.5, 2.1); 7.65 (H2', d J = 2.1)
Lathyrus cicera	Kaempferol derivative 1	6.52 (H6, d, J = 2.1); 6.78 (H8, d, J = 2.1); 7.06 (H2'/H6', d, J = 8.4); 8.02 (H3'/H5', d, J = 8.4)
	L-homoarginine	1.63 (H6, m); 1.87 (H4, m); 3.20 (H7, t)
	γ -pyrone	6.69 (d, J=5.1); 8.17 (d, J=5.1)
Lathyrus clymenum	Kaempferol	see *A. boeticus*
	Catechin	2.52 (H4b, dd); 2.82 (H4a, dd); 5.95 (H6, d, J = 2.1 Hz); 6.03 (H8, d, J = 2.1); 6.94–6.96 (ring B, ov)
Medicago minima	Daidzein	6.96 (H3'/H5', d J = 9.0); 6.97 (H8, d J = 2.0); 7.04 (H6, dd J = 9.0, 2.0); 7.43 (H2'/H6', d J = 9.0); 8.06 (H5, d J = 9.0); 8.21 (H2, s)
	Daidzin	5.24 (H1'', d, J = 7.8); 6.96 (H3'/H5', d J = 9.0); 7.28 (H8, d J = 2.0); 7.25 (H6, dd J = 9.0, 2.0); 7.41 (H2'/H6', d J = 9.0); 8.17 (H5, d J = 9.0); 8.29 (H2, s)
	Genistein	6.31 (H6, d J = 2.0), 6.47 (H8, d J = 2.0), 7.05 (H3'/H5', d J = 9.0); 7.44 (H2'/H6', d J = 9.0); 8.21 (H2, s)
	Phenylpropanoid	6.42 (H8, d, J = 15.9) 7.60 (H7, d, J = 15.9)
	Quercetin-7-O glucoside	5.23 (H1'', d, J = 7.8); 6.54 (H6, d, J = 2.1); 6.90 (H8, d, J = 2.1); 7.06 (H5', d, J = 8.4); 7.66 (H6' , dd, J = 8.7, 2.1); 7.83 (H2', d, J = 2.1)
	Trigonelline	9.15 (H1, s); 8.86 (H3/H5, t)
Melilotus neapolitanus	Coumarin	6.51 (H3, d J = 9.6); 8.08 (H4, d J = 9.6); 6.89 (H6/H8, m); 7.40 (H5/H7, m)
	cis-Melilotoside	5.03 (H10, d J = 7.8); 6.06 (H3, d J = 12.6); 6.86 (H4, d J = 12.6)
	trans-Melilotoside	5.01 (H10, d J = 7.5); 6.84(H3, d J = 16.2); 7.86 (H4, d J = 16.2);
	Trigonelline	see *M. minima*
Ononis variegata	Caffeic acid derivative 1	6.47 (H-8′, d, J = 15.9 Hz); 6.88 (H-5′, d, J = 8.2 Hz); 7.09 (H6',dd, J=8.4, 2); 7.18 (H2', d, J=2.1); 7.67 (H7, d, J=15.9)
	Quercetin derivative 1	6.32 (H6, d, J = 2.1); 6.53 (H8, d, J = 2.1); 6.95 (H5', d J = 8.5); 7.55 (H6', d J = 8.5, 2.1); 7.66 (H2', d J = 2.1)
Pisum sativum	Flavonoids	6.31 (ring A, d, J = 2.1); 6.52 (ring A, d, J = 2.1)
Trifolium campestre	C6-glycosilated apigenin derivative	6.72 (H8, S); 6.91(H8, S); 7.10 (H3'/H5',d, J = 9.0 Hz); 7.93 (H2'/H6',d, J = 9.0 Hz)
	Trigonelline	see *M. minima*
Trifolium cherleri	Caffeic acid derivative 2	7.61 (H7, d, J=15.9); 7.18 (H2', d, J=2.1); 7.07 (H6',dd, J=8.4, 2); 6.87 (H-5′, d, J = 8.2 Hz); δ 6.39 (H-8′, d, J = 15.9 Hz)
	Kaempferol derivative 2	6.32 (H6, d, J = 2.1); 6.53 (H8, d, J = 2.1); 6.95 (H2'/H6', d, J = 8.4); 8.17 (H3'/H5', d, J = 8.4)
	Quercetin derivative 2	6.27 (H6, d, J = 2.1); 6.52 (H8, d, J = 2.1); 6.95 (H5', d J = 8.5); 7.55 (H6', dd, J = 8.5, 2.1); 7.61 (H2', d J = 2.1)

	Trigonelline	see *M. minima*
Trifolium scabrum	Daidzein	see *M. minima*
	Daidzin	see *M. minima*
	Genistein	see *M. minima*
	Quercetin derivative 3	6.27(H6, d, J = 2.1); 6.46 (H8, d, J = 2.1); 6.92 (H5', d J = 8.5); 7.52 (H6', d J = 8.5, 2.1); 7.58 (H2', d J = 2.1)
	Kaempferol derivative 3	6.28 (H6, d J = 2.1), 6.48 (H8, d J = 2.1), 6.93 (H3'/H5', d J = 8.5); 7.96 (H3'/H5', d, J = 8.5)
Trigonella esculenta	Caffeic acid derivative 3	7.59 (H7, d, J=15.9); 7.15 (H2', d, J=2.1); 7.06 (H6',dd, J=8.4, 2); 6.86 (H-5′, d, J = 8.2 Hz); δ 6.38 (H-8′, d, J = 15.9 Hz)
	4-hydroxyisoleucine	0.98 (H6, d, J=6.8); 1.25 (H5, d, J=6.3)
	Quercetin derivative 4	6.27 (H6, d, J = 2.1); 6.53 (H8, d, J = 2.1); 6.97 (H5', d J = 8.5); 7.67 (H6', dd, J = 8.5, 2.1); 7.75 (H2', d J = 2.1)
	Trigonelline	see *M. minima*
Vicia bithynica	Chlorogenic acid	6.37 (H8', d, J=15.9); 7.61 (H7', d, J=15.9); 7.14 (H5', d, J = 2.0 Hz); 7.06 (H6', d, J = 8.5; 2.2 Hz) 6.88 (d, J = 8.2); 1.84 - 2.20 (H2/H6, m); 5.45 (ring A, ov)
	Quercetin	See *A. boeticus*
	Quercetrin	0.91 (H6'', d, J = 6.0); 5.27 (H1'', d, J = 1.8); 6.31 (H6, d, J = 2.1); 6.51 (H8, d, J = 2.1); 6.98 (H5', d, J = 8.4); 7.34 (H6' , dd, J = 8.7, 2.1); 7.37 (H2', d, J = 2.1)
Vicia pseudocracca	Quercetrin	see *V. bithynica*
	Trigonelline	see *M. minima*
Vicia angustifolia	Chlorogenic acid	see *V. bithynica*
	Quercetin derivative 5	6.31 (H6, d, J = 2.1); 6.56 (H8, d, J = 2.1); 7.00 (H5', d J = 8.5); 7.64 (H6', dd, J = 8.5, 2.1); 7.67 (H2', d J = 2.1)
